# Impact of early pericardial fluid chymase activation after cardiac surgery

**DOI:** 10.3389/fcvm.2023.1132786

**Published:** 2023-04-12

**Authors:** Brittany Butts, Lee A. Goeddel, Jingyi Zheng, Betty Pat, Pamela Powell, James Mobley, Sarfaraz Ahmad, Chad Steele, David McGiffin, James E. Davies, James F. George, Spencer J. Melby, Carlos M. Ferrario, Louis J. Dell’Italia

**Affiliations:** ^1^ Nell Hodgson Woodruff School of Nursing, Emory University, Atlanta, GA, United States; ^2^ Department of Anesthesia and Critical Care Medicine, Johns Hopkins University, Baltimore, MD, United States; ^3^ Department of Mathematics and Statistics, College of Science and Mathematics, Auburn University, Auburn, AL, United States; ^4^ Division of Cardiovascular Disease, Department of Medicine, The University of Alabama at Birmingham (UAB), Birmingham, AL, United States; ^5^ Department of Veterans Affairs, Birmingham Veterans Affairs Health Care System, Birmingham, AL, United States; ^6^ Department of Anesthesiology and Perioperative Medicine, University of Alabama at Birmingham (UAB), Birmingham, AL, United States; ^7^ Department of Surgery, Wake Forest School of Medicine, Winston-Salem, NC, United States; ^8^ School of Medicine—Microbiology and Immunology, Tulane University, New Orleans, LA, United States; ^9^ Cardiothoracic Surgery and Transplantation, The Alfred Hospital, Monash University, Melbourne, VIC, Australia; ^10^ Department of Surgery, Division of Cardiothoracic Surgery, University of Alabama at Birmingham (UAB), Birmingham, AL, United States; ^11^ Department of Surgery, Division of Cardiothoracic Surgery, Washington University, Saint Louis, MO, United States; ^12^ Saint Louis VA Medical Center, Birmingham VA Health Care System, Birmingham, AL, United States

**Keywords:** chymase, extracellular vesicles, exosomes, cardiovascular surgery, inflammation, length of stay, STS-PROM, pericardial fluid

## Abstract

**Introduction:**

Chymase is a highly destructive serine protease rapidly neutralized in the circulation by protease inhibitors. Here we test whether pericardial fluid (PCF) chymase activation and other inflammatory biomarkers determine intensive care unit length of stay, and explore mechanisms of chymase delivery by extracellular vesicles to the heart.

**Methods:**

PCF was collected from adult patients (17 on-pump; 13 off-pump) 4 h after cardiac surgery. Extracellular vesicles (EVs) containing chymase were injected into Sprague–Dawley rats to test for their ability to deliver chymase to the heart.

**Results:**

The mean intensive care unit (ICU) stay and mean total length of stay was 2.17 ± 3.8 days and 6.41 ± 1.3 days respectively. Chymase activity and 32 inflammatory markers did not differ in on-pump vs. off-pump cardiac surgery. Society of Thoracic Surgeons Predicted Risk of Morbidity and Mortality Score (STS-PROM), 4-hour post-surgery PCF chymase activity and C-X-C motif chemokine ligand 6 (CXCL6) were all independent predictors of ICU and total hospital length of stay by univariate analysis. Mass spectrometry of baseline PCF shows the presence of serine protease inhibitors that neutralize chymase activity. The compartmentalization of chymase within and on the surface of PCF EVs was visualized by immunogold labeling and transmission electron microscopy. A chymase inhibitor prevented EV chymase activity (0.28 fmol/mg/min vs. 14.14 fmol/mg/min). Intravenous injection of PCF EVs obtained 24 h after surgery into Sprague Dawley rats shows diffuse human chymase uptake in the heart with extensive cardiomyocyte damage 4 h after injection.

**Discussion:**

Early postoperative PCF chymase activation underscores its potential role in cardiac damage soon after on- or off-pump cardiac surgery. In addition, chymase in extracellular vesicles provides a protected delivery mechanism from neutralization by circulating serine protease inhibitors.

## 1. Introduction

Operative trauma, cardiopulmonary bypass (CPB), and reperfusion injury leads to a robust systemic inflammatory response (1). In the heart, resident mast cells and neutrophil infiltration play an important role in reperfusion injury (2, 3). Recruitment of mast cells and neutrophils to the site of cardiac injury leads to the *de novo* production of inflammatory chemokines and cytokines, such as TNF-α (4, 5). In addition, mast cells release granules that contain serine proteases such as elastase, cathepsin G, and chymase (6, 7). Recent studies have demonstrated the beneficial effects of mast cell stabilizers in cardiac protection from ischemia/reperfusion injury (8–10). The activation of mast cell proteolytic enzymes are the primary cause of tissue damage at the site of myocardial injury, in large part through activation of matrix metalloproteinases (5). Furthermore, recruitment of neutrophils to the injured site is coordinated by chemokines such as C-X-C motif chemokine ligand 6 (CXCL6) that establish chemotactic gradients between the blood and tissue. Both mast cells and neutrophils release chemokines and serine proteases upon degranulation, leading to a positive feedback loop of sustained inflammatory response (11).

We recently demonstrated a robust augmentation in chymase activity, the acute phase proteins tumor necrosis factor-alpha (TNF-α), CXCL6, and myeloperoxidase (MPO), in the PCF of patients undergoing cardiac surgery as early as 4 h after completing the procedure (12). The activation of integral components of the innate immune response is associated with an increased presence of PCF neutrophils (13). The presence of neutrophils explains the significant increase in myeloperoxidase, neutrophil gelatinase-associated lipocalin (NGAL), and CXCL6 (12). Infiltration of inflammatory cells is accompanied by increases in oxidative stress, as demonstrated by elevated concentrations of reactive oxygen species, oxidized hemoglobin, and oxidized phospholipids in PCF (13).

The serendipitous discovery that the contents of extracellular vesicles could be extruded by cells to effect the biological response of recipient cells (14–16) has attracted major attention because these extracellular vesicles [(EVs); 50–1000 nm] and cell-derived exosomes [(EXOs); 40–160 nm] contribute to the mechanisms accounting for immune responses, viral pathogenicity, chronic diseases of the heart, blood vessels, and kidney, and oncogenic processes (16). An emerging literature reveals a critical role of EXOs in the homeostatic control of myocardial function and adverse cardiac remodeling (17). Novel roles of cardiac EXOs in conveying regulatory actions upon the expression of a myocardial renin angiotensin system (RAS) was provided by Lyu and colleagues (18). In this study angiotensin II (Ang II) stimulated the release of neonatal rat cardiac fibroblast EXOs and increased Ang II production (19). These findings and those of Bang et al.(19) reveals the existence of a paracrine mechanism in which EXOs modulate the pathological actions of a locally functioning RAS (20).

The importance of chymase as a destructive protease (5) as well as the primary hydrolytic pathway for tissue Ang II generation from angiotensin I (Ang I) (21–23) and the novel angiotensinogen substrate -angiotensin-(1‒12) [Ang-(1‒12)] (24–26) led us to undertake a comprehensive characterization of human PCF chymase activity, inflammatory cytokines, and EV cargo following open-cardiac surgery. The functional window afforded by serial collections of PCF provides a unique opportunity to monitor and precisely document the adaptive cardiac response to the tissue insult associated with cardiac surgery in humans. In addition, the translational impact of this study gained robustness by correlating for the first time the impact of the locally generated immuno-mediated inflammatory response to a clinical endpoint of operative mortality and morbidity risk as calculated by the STS-PROM pre-operative risk score established by the Society of Thoracic Surgeons (27, 28).

## 2. Material and methods

### 2.1. Patient population and pericardial fluid collection

This study includes a cohort of 30 patients (23% females) undergoing cardiac surgery for coronary artery bypass grafting (CABG), valvular heart disease, or combined valvular surgery with CABG (Table 1). Patients with ventricular assist devices, atrial fibrillation and re-do surgery, thoracic aorta surgery, concomitant non-cardiac surgery, and on pre-operative inotropic support were excluded from the study. The study conforms to the ethical guidelines of the 1975 Declaration of Helsinki, and was approved by the Institutional Review Board at the University of Alabama at Birmingham. All participants provided written informed consent. PCF was collected directly on the opening of the pericardial sac (time 0) and then from pericardial drains at 4, 12, and 24 h after surgery, as described previously by our lab (12, 13).

**Table 1:** Demographic and clinical characteristics.

N = 30	Mean ± SD	Range
	N	%
Age (years)	63 ± 2	38–78
BMI (kg/m^2^)	29 ± 1	19–51
Gender		
Female	7	23%
Male	23	77%
Race		
Black	5	17%
White	25	83%
Surgery		
CABG	20	67%
Valve Repair	5	17%
CABG + Valve	5	17%
Cardiopulmonary Bypass		
OFF	13	43%
ON	17	57%
Pre-operative LVEF (%)	50 ± 11	15–55
Beta blocker	18	60%
ACEi/ARB	14	47%
Statin	16	53%
Aspirin	17	57%

ACEi, angiotensin converting enzyme inhibitor; ARB, angiotensin II receptor blocker; BMI, body mass index; CABG, coronary artery bypass graft; LVEF, left ventricular ejection fraction.

STS-PROM pre-operative risk score was performed using the Online STS Adult Cardiac Surgery Risk Calculator at http://riskcalc.sts.org/stswebriskcalc/#/. The STS Risk Calculator is used in clinical decision-making and for communicating the individualized expected risk of cardiac surgery with patients and caregivers (27, 28).

### 2.2. Primary outcome: Intensive care unit and total hospital length of stay

All patients were admitted to the cardiac surgery intensive care unit (ICU) from the operating room and arrival time in the ICU was designated as time zero. For ICU length of stay, follow-up ended on the day the patient was transferred to a lower acuity care setting. No patients in this study were readmitted to the intensive care setting. The total length of stay was calculated as days from surgery to the date of discharge.

### 2.3. Biomarker measurement

The Luminex fluorescent bead-based multiplex assay in a 96-well format (Milliplex kit, Millipore Corp.) was used to measure PCF biomarkers at 4 h, according to manufacturer instructions, as previously described in our lab (12, 13). Troponin-1 and brain natriuretic peptide (BNP) were measured as metrics of cardiac injury, TNF-α and chymase as products of mast cells, and myeloperoxidase and neutrophil gelatinase-associated lipocalin (NGAL) were measured as products of neutrophils. Standard curves were generated for each analyte using the standards supplied by the manufacturer to correlate mean fluorescence intensity with concentration.

Radiolabeled (^125^I) angiotensin-(1–12) [Ang-(1–12)] was used as a substrate for the determination of chymase activity, as previously described (24, 25). Briefly, PCF was pre-incubated (10 min) in the presences or absence of the chymase inhibitor-chymostatin (50 μM) in assay buffer. Additional inhibitors (lisinopril for angiotensin-converting enzyme [ACE], SCH39373 for neprilysin, MLN-4760 for ACE2, amastatin and bestatin for aminopeptidases, benzyl succinate for carboxypeptidases and *ρ*-chloromercuribenzoic acid [PCMB] for cysteine proteases, each 50 μM) were added to inhibit the enzymatic activities of the respective enzymes (26). Following preincubation of PCF with the inhibitor cocktail, radiolabeled ^125^I-Ang-(1–12) substrate was added to the reaction and incubated for 2 h at 37°C. An equal volume of 1% phosphoric acid was added to stop the reaction followed by centrifugation at 28,000 *g* for 20 min. Clear supernatants were filtered (0.2 μm PVDF membrane) and injected onto a HPLC C-18 column. Chymase generated ^125^I-Ang II from ^125^I-Ang-(1–12) was detected with an in-line flow-through gamma detector (BioScan Inc., Washington, DC). Chymase activity was defined as fmol of Ang II product formed from ^125^I-Ang-(1–12) substrate/mL/minute (fmol Ang II formation/ml/min). The content of angiotensin II in PCF was measured by radioimmunoassay, as described previously (29, 30).

For western blot analysis, 20 μl of pericardial fluid (diluted 1:10 in PBS) added to an equal volume of 2x NuPAGE™ LDS Sample Buffer containing NuPAGE™ sample reducing agent (Invitrogen, Thermo Fisher Scientific Co, Waltham, MA) and denatured at 95°C for 5 min was separated on (10 μg of protein) on a NuPAGE™ Novex 4%–12% Bis-Tris, transferred to a 0.2 µm PVDF membrane (Invitrogen), and probed with anti-human chymase/CMA1 antibody (mouse monoclonal; 1:500; R&D Systems Inc. Minneapolis, MN). Membranes were washed in PBS/0.1% Tween 20 and then incubated in horseradish peroxidase (HRP)-conjugated secondary antibodies (Bio-Rad Laboratories Inc., Hercules CA; 1:2,000–1:5,000). HRP signals were developed using Clarity Western enhanced chemiluminescence substrate (Bio-Rad Laboratories) on a FluorChem M system (Protein Simple).

### 2.4. PCF extracellular vesicle isolation

Extracellular vesicles were isolated from the pericardial space upon opening of the pericardium and these EVs were used for transmission electron microscopy Immunogold studies. Extracellular vesicles were isolated from the pericardial drain at 24 h and these EVs were used for injection into rats. Extracellular vesicles isolated from PCF (4–6 ml), were pelleted by differential ultracentrifugation (DUC). PCF was centrifuged at 4,000 × g for 10 min at 4°C (spin 1) to pellet cells and other larger particles/debris, then centrifugation at 20,000 × g for 1 h at 4°C to pellet microvesicles (spin 2), and a final centrifugation at 150,000 × g for 1.5 h at 4°C to pellet exosomes (spin 3) (31, 32). EVs were diluted 1:4–1:10 in Measurement Electrolyte (Izon Science Ltd) to measure the size (nm) and concentration (particles/mL) (32). EVs were used immediately or stored for up to 7 days at 4°C prior to use. EV chymase activity was measured with the fluorescent substrate N-Succinyl-Ala-Ala-Pro-Phe-7-amido-4 methylcoumarin (Sigma-Aldrich, St. Louis MS; S9761) as described previously (33).

### 2.5. Extracellular vesicle transmission electron microscopy and immunogold labeling

EV pellets (after spin 2 and 3) from PCF at the time of opening the pericardium were suspended in 0.1 M sodium cacodylate buffer pH 7.41 (NaCaC) for 1–2 h at 4°C to fix pellets and processed for transmission electron microscopy (TEM) as previously described in our lab (32). For immunogold labeling, EV pellets were submerged in 0.5% glutaraldehyde 3% paraformaldehyde in buffer (5% dextrose, 30 mM potassium chloride in PBS), further fixed in the same fixative in cacodylate buffer, incubated in 0.1M glycine/PBS, dehydrated in a series of N, N-dimethyl formamide, and embedded in LR White resin. Ultrathin (90 nm) sections were picked up onto nickel grids (3 mm), dried, and etched in a 3% solution of sodium m-periodate in 0.1N HCl (2 × 30 min each). Prior to immunolabeling, grids were rinsed in PBS (3 x) and blocked for 1.5 h with 1% BSA/1% goat serum/0.1% cold water fish skin gelatin/0.1% tween-20 in PBS. Grids were incubated with primary antibodies (diluted 1:25 in 1% BSA/PBS) to mouse monoclonal human CD9 (EMD Millipore #CBL162), rabbit polyclonal Annexin V (GeneTex #GTX103250), mouse monoclonal [CC1] to human mast cell chymase (Abcam #ab2377) or rabbit monoclonal [EPR13136] to recombinant mast cell chymase (Abcam #ab186417), for 2 h at room temperature and again overnight at 4°C. Grids were rinsed with PBS (4 x), blocked in 1% BSA/1% goat serum/PBS for 1.5 h, and incubated for 2 h at room temperature with goat anti-rabbit or anti-mouse immunoglobulin G tagged with colloidal gold (10-nm or 15-nm particle size respectively, diluted 1:50 with 1% BSA/PBS) (Aurion/Electron Microscopy Sciences, Hatfield, PA). Following post-fixation and counterstain with uranyl acetate, sections were imaged on a FEI-Tecnai T12 Spirit 20 electron microscope at 120 kv as previously described in our laboratory (32).

### 2.6. Mass spectroscopy of PCF content

PCF isolated from two patients (Patient 06 and 16) at opening of the pericardium (time 0) were assessed by BCA protein assay (Thermofisher, Waltham, MA), and 40 µg were run in triplicate on a 10% Bis-Tris gel, and stained with Colloidal Coomassie Stain (Invitrogen, Waltham, MA) overnight. The molecular weight corresponding to 27–34 kDa range was trypsin digested. Digests from all three lanes (for each sample) were combined prior to LC-MS analysis (Supplementary Figure S1).

### 2.7. Extracellular vesicle injection in rats

EVs isolated from the PCF drain at 24 h after surgery were intravenously injected in a single bolus (109–10 particles) into the jugular vein of adult male Sprague-Dawley rats as previously described in our laboratory (31, 32). Rats were euthanized at 4 and 24 h after injection, and heart tissue was excised, snap-frozen in liquid nitrogen, or fixed in formalin. Immunohistochemistry was performed on 5 µm formalin fixed paraffin embedded tissue sections using mouse anti-human mast cell chymase CC1 (1:50, Abcam ab2377), and rabbit anti-von Willebrand Factor (1:150, Chemicon 7356). Alexa Fluor 488- and 594-conjugated secondary antibodies (1:700; Life Technologies/Invitrogen Eugene, OR) were used and nuclei (blue) were counterstained with 4,6-diamidino-2-phenylindole (DAPI, 1.5 μg/ml; Vector Laboratories, Burlingame, CA). Images were acquired on a Leica DM6000 epifluorescence microscope with Simple-PCI software (Compix, Cranberry Township, PA) and adjusted appropriately to minimize background fluorescence.

### 2.8. Statistical analysis

Data in Tables are presented as mean ± standard deviation or counts (percent) for continuous variables and categorical variables respectively. The Mann-Whitney-Wilcoxon test was used for between-group comparisons. *P* values were adjusted for multiple testing *via* false discover rate (FDR)-controlling procedure in Supplementary Table S1 in comparing biomarkers for on- and off-pump cardiac surgery. For the length of stay analysis, a zero-truncated Poisson regression model was constructed to estimate the effect of a predictor variable. Truncation at zero accounts for the actuality that all post-cardiac patients will not spend zero days in the ICU and hospital, which is applicable in this patient population. The variable coefficients in this model represent the difference in the logs of the expected outcome variable (i.e., length of stay) per unit change in the predictor variables. Clinically, a coefficient that is significantly different from zero indicates an increase (if positive) or decrease (if negative) in the expected length of stay for every unit increase in the predictor variable, while controlling for other variables in the model.

Univariate models were fit to examine the individual biomarker effect, and multivariate models were fit to control for STS-PROM. To compare model performance, we obtained the model fit statistics, including Akaike Information Criterion (AIC), corrected AIC for small sample (AICC), and Bayesian Information Criterion (BIC). All multivariable models included only two variables: STS-PROM and a biomarker. Data were analyzed with SAS software version 9.4 with an alpha set at 0.05.

Data (continuous variables) in figures are presented as box (lower and upper quartiles) and whisker (minimum and maximum values) plots with the median (horizontal line in box). The mean value is indicated by the red diamond. Each symbol is either a patient or an animal.

## 3. Results

### 3.1. Patient demographics

The study population was predominately male and Caucasian (Table 1). Most participants underwent a coronary artery bypass grafting procedure (67%). Over half of the study group were on-pump cardio-pulmonary bypass (CPB) surgeries (57%). ICU length of stay ranged from one to 21 days, with a mean of 2.17 ± 3.8 days. The total length of stay ranged from 3 to 40 days, with a mean of 6.41 ± 1.3 days.

Mean STS-PROM and 4-hour chymase activity, chemokine/cytokine, troponin, and BNP values are listed in Table 2. There were no differences in ICU or total length of stay related to gender, race, or CPB status (*p* > 0.05 for all). STS-PROM was higher in participants who underwent any valve procedure as compared to a CABG procedure alone (12.83 ± 6.4 vs. 5.12 ± 1.1, respectively, *p* < 0.001). There were no differences in biomarkers between surgery groups or between on- and off-pump surgeries (Supplementary Table S1). Intraoperative cross-clamp time and total bypass time were positively related to STS-PROM (*r* = 0.930, *p* = 0.0003 and *r* = 0.890, *p* = 0.001, respectively) and chymase (*r* = 0.637, *p* = 0.004 and *r* = 0.590, *p* = 0.01, respectively), but no other 4-hour PCF biomarkers.

**Table 2:** STS-PROM and pericardial fluid markers (4 h after surgery).

N = 30	Mean ± SD	Min–Max
STS-PROM	7.51 ± 3.6	3.2–28.3
Troponin-1 (µg/ml)	0.15 ± 0.08	0.06–0.24
BNP (pg/ml)	259.84 ± 132.1	93.4–468.2
Chymase activity (fmol/ml/min)	8.76 ± 2.5	2.4–21.6
TNF-α (pg/ml)	6.15 ± 1.1	1.4–16.9
Myeloperoxidase (pg/ml)	550.61 ± 137.7	55.2–2226.2
Interleukin-8 (pg/ml)	66.74 ± 29.0	1.7–427.2
NGAL (pg/ml)	119.81 ± 17.9	36.6–553.0
CXCL6 (pg/ml)	8.67 ± 1.3	2.8–20.4

BNP, brain natriuretic peptide; CXCL, chemokine (C-X-C motif) ligand; NGAL, neutrophil gelatinase-associated lipocalin; STS-PROM, Society of Thoracic Surgeons Predicted Risk of Morbidity and Mortality; TNF, tumor necrosis factor.

### 3.2. Predictors of length of stay

The model fitting results are summarized in Supplementary Table S2 (prediction of length of hospital stay) and Supplementary Table S3 (prediction of ICU stay). *P* values in the tables indicate the significance of each predictor variable. The predictor variables of interest to model the length of stay were STS-PROM, troponin-1, BNP, chymase activity, TNF-α, myeloperoxidase, Interleukin-8, NGAL, and CXCL6. Univariate Poisson regression demonstrates that STS-PROM and 4-hour PCF chymase activity, TNF-α, myeloperoxidase, and CXCL6 are significant predictors of total hospital length of stay (Table 3). Multivariable Poisson regression models with 4-hour PCF chymase activity plus STS-PROM as predictor variables demonstrate best model fitting for prediction of total hospital length of stay with the lowest model fit statistics (AIC/AICC/BIC: 72.7/74.7/75.0) (Table 3).

**Table 3:** Zero truncated poisson regression relating pericardial fluid markers (at 4 h after surgery) and STS-PROM score to hospital length of stay.

Model	Coefficient	SE	*p*-value	AIC/AICC/BIC
Univariate Analysis				
STS-PROM	0.093	0.009	<.0001	156.6/157.7/159.4
Chymase (fmol/ml/min)	0.127	0.014	<.0001	117.2/117.7/119.8
CXCL6 (pg/ml)	0.009	0.0001	<.0001	179.2/179.7/181.9
TNF-α (pg/ml)	0.0026	.0015	.0026	200.1/200.7/202.6
Myeloperoxidase (pg/ml)	0.0001	0.00002	<.0001	156.8/157.6/158.6
Multivariate Analysis				
STS-PROM+	0.051	0.021	.0132	72.7/74.7/75.0
Chymase (fmol/ml/min)	0.068	0.033	.04	
STS-PROM+	0.06	0.02	.0001	88.9/90.4/92.1
BNP (pg/ml)	0.002	0.0007	.01	
STS-PROM+	0.080	0.009	<.0001	110.1/111.1/114.0
CXCL6 (pg/ml)	0.006	0.0002	.0016	
STS-PROM+	0.092	0.009	<.0001	108.7/109.9/112.2
TNF-α (pg/ml)	0.001	0.002	.05	

BNP, brain natriuretic peptide; CXCL, chemokine (C-X-C motif) ligand; STS-PROM, Society of Thoracic Surgeons Predicted Risk of Morbidity and Mortality; TNF, tumor necrosis factor; AIC, Akaike Information Criterion; AICC, corrected AIC for small sample; and BIC, Bayesian Information Criterion.

ICU length of stay univariate models demonstrates that STS-PROM and 4-hour PCF chymase activity, myeloperoxidase, and CXCL6 are significant predictors (Table 4). Again, the multivariable Poisson regression that includes STS-PROM plus 4-hour PCF chymase activity has the best model fitting for the prediction of ICU length of stay (AIC/AICC/BIC: 30.2/32.2/32.5) (Table 4).

**Table 4:** Zero truncated poisson regression relating pericardial fluid markers (at 4 h after surgery) and STS-PROM score to intensive care unit length of stay.

Model	Coefficient	SE	*p*-value	AIC/AICC/BIC
Univariate Analysis				
STS-PROM	0.175	0.020	<.0001	71.3/71.8/74.0
Chymase (fmol/ml/min)	0.241	0.028	<.0001	46.7/47.2/49.3
CXCL6 (pg/ml)	0.002	0.0002	<.0001	105.9/106.4/108.5
Multivariate Analysis				
STS-PROM+	0.009	0.067	0.89	30.2/32.2/32.5
Chymase (fmol/ml/min)	0.271	0.130	0.04	
STS-PROM+	0.139	0.022	<.0001	44.64/45.7/48.5
CXCL6 (pg/ml)	0.001	0.0005	.03	

CXCL, chemokine (C-X-C motif) ligand; STS-PROM, Society of Thoracic Surgeons Predicted Risk of Morbidity and Mortality; AIC, Akaike Information Criterion; AICC, corrected AIC for small sample; and BIC, Bayesian Information Criterion.

### 3.3. Chymase activity and angiotensin ll levels in PCF

**Figure 1:**
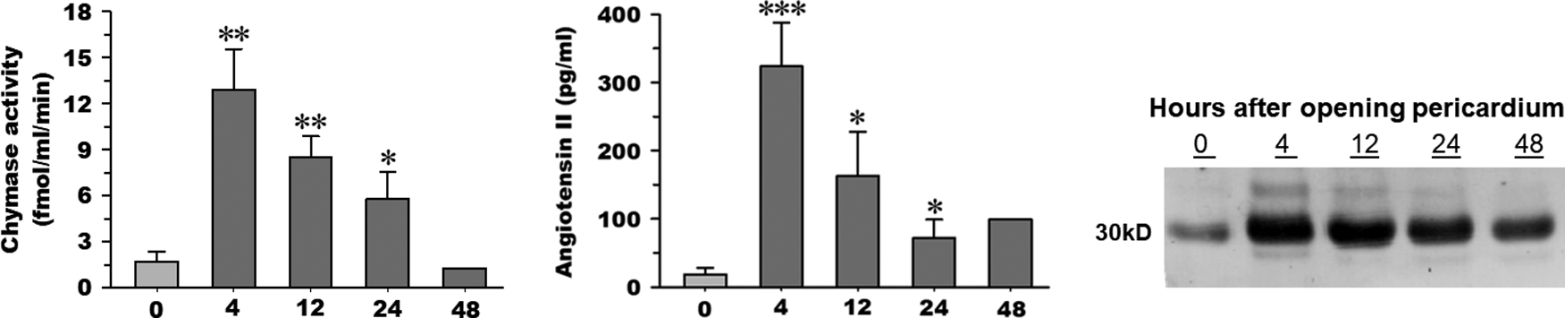
Chymase activity (**A**) and angiotensin II peptide concentration (**B**) measured in pericardial fluid (*n* = 5) upon opening of the pericardium (time 0), 4, 12, and 24 h time points after cardiopulmonary bypass. Chymase activity is calculated based on chymase-mediated Ang-(1–12) metabolism. Representative western blot (**C**) of human chymase in an equal volume of PCF obtained from the same patient (1 of the 5) at documented time points in the other panels (time 0, 4, 12, and 24 h after cardiopulmonary bypass). 05; **p < 0.01; ***p < 0.001 vs. time 0. Box and whisker plots (median, interquartile range with min and max values). Mean indicated by red diamond).

Chymase generates angiotensin II at a more efficient and greater rate than angiotensin-converting enzyme (ACE) (21–23, 34). Both chymase activity and Ang II are significantly elevated in PCF at 4 h post-cardiac surgery (Figures 1A,B) and remain higher than baseline through 24 h after surgery. PCF chymase protein demonstrates a similar pattern, increasing at 4 h and remaining above baseline 24 h after surgery (Figure 1C).

### 3.4. Chymase in extracellular vesicles

**Figure 2:**
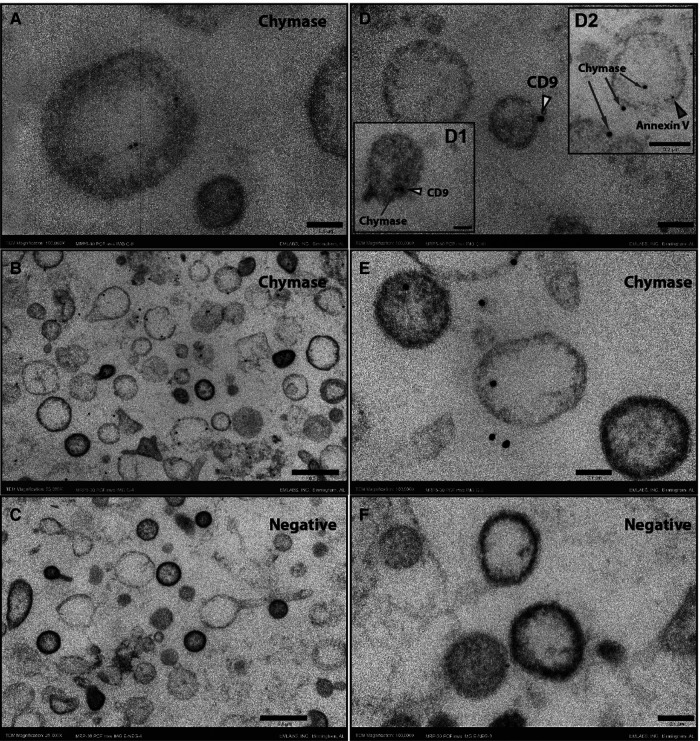
Immunogold ultrathin transmission electron microscopy of microvesicles at time 0. Immunogold TEM using gold beads bound to chymase, CD9 or Annexin V antibodies. Chymase is found inside vesicles (**A, D2, E**) but is predominantly on the membrane surface (**B, D1, D2, E**) as indicated by co-labeling for the tetraspanin CD9 (yellow arrowheads) or Annexin V (red arrowhead). The omission of the chymase antibody shows no staining in the negative sections (**C,F**). Scale bars 0.5 *μ*m - 25,000X (**B, C**), 0.2 *μ*m - 50,000X (**inset D2**) and 0.1 μm -100,000X (**A, D, inset D1, E,F**).

Although blood serine protease inhibitors (SERPINs) readily inhibit circulating chymase, similar observations have not been reported previously in PCF. Examination of the PCF at the time of pericardiectomy by liquid chromatography mass spectrometry demonstrates a significant presence of *α*1-anti-trypsin and *α*1-anti-chymotrypsin, serine protease inhibitors in the human (Supplementary Table S4, Supplementary Figure S1). To resolve the paradox of high chymase enzymatic activity and protein in the presence of endogenous chymotrypsin inhibitors we explore the location of the enzyme in the cellular elements present in the PCF. TEM with immunogold staining shows that chymase is housed within EVs and therefore likely protected from neutralization. Figure 2 demonstrates chymase on the surface and within PCF EVs (exosomes and microvesicles) by immunogold TEM staining. Double immunogold staining with Annexin V and CD9 identify chymase with extracellular vesicles. Western blot of PCF extracellular vesicles from five patients at the time of opening the pericardium, shows chymase in all samples (Supplementary Figure S2).

### 3.5. Uptake of human chymase in rat heart and lung after injection of PCF EVs

**Figure 3:**
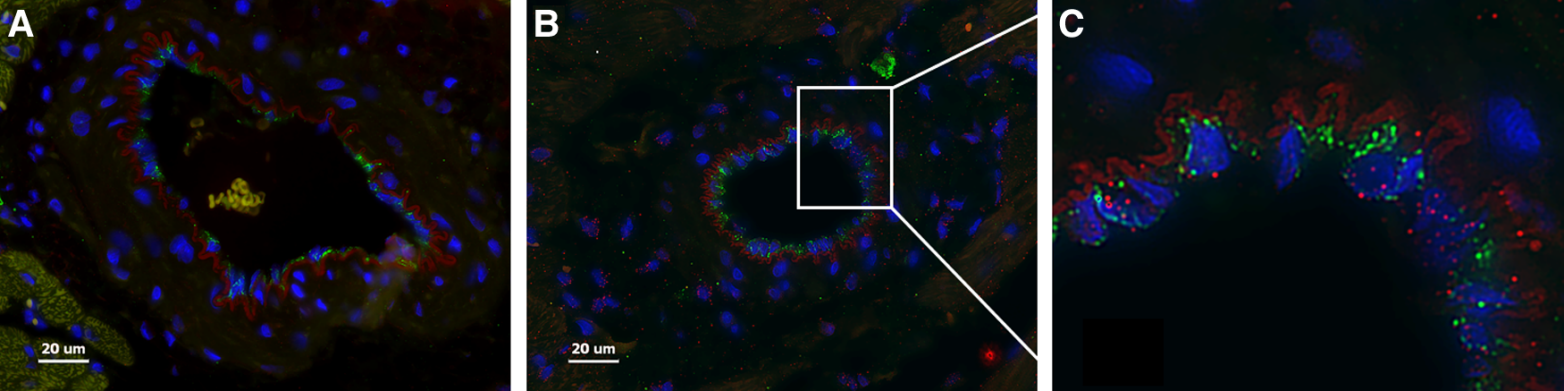
Naïve saline injected rat (**A**) and a rat 4 h after injection with PCF microvesicles obtained from a patient (24 h after cardiac surgery) demonstrate human chymase (red) (**B, C**) in the interstitium and within endothelial cells (**B**-**white box inset of C**) in the rat heart. DAPI nuclei: Blue, von Willebrand Factor: Green (endothelium). The red outline around the vessel is the autofluorescence of the internal elastic membrane. Red blood cells auto fluoresce with a subdued yellowish tinge (within a vessel in A). Scale bar = 20 µm.

**Figure 4:**
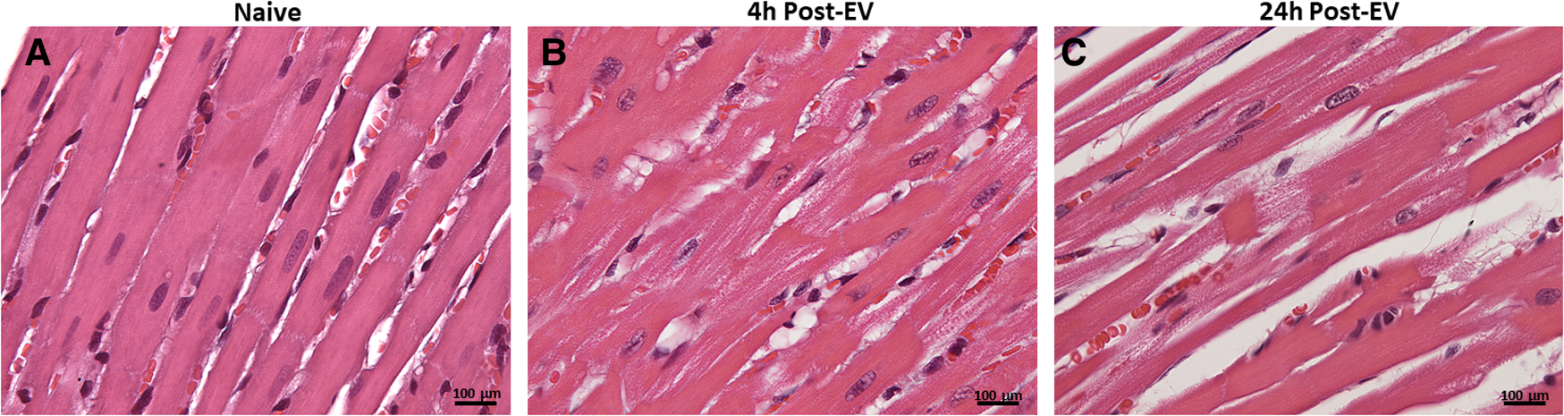
H&E staining of naïve saline injected rat heart (**A**) and four and 24 h after injection of extracellular vesicles (**B,C**). The normal heart (**A**) demonstrates homogeneous H&E staining and compactly spaced cardiomyocytes. Microvesicle-injected hearts demonstrate increased interstitial space and diffuse breakdown of cardiomyocytes and myosin (**B, 20X**). At 40x, there is myocyte loss, myosin breakdown, and damaged capillary and endothelial cells (**arrow**) consistent with the increased endothelial chymase in (**C**).

Here we present delivery of human chymase *via* extracellular vesicles to rat heart and lung (Figure 3) after a single bolus intravenous injection of ∼109–10 microvesicles isolated from the PCF drain 24 h after cardiac surgery in a single patient. These microvesicles have chymase activity (14.14 fmol/mg/min) that is significantly inhibited (0.28 fmol/mg/min) by the chymase inhibitor–TEI-F00806 (Teijin Pharma Ltd, Tokyo, Japan). We have previously demonstrated that a human chymase antibody does not interact with rat mast cell proteases (33), thus taking advantage of the ability to detect human chymase within rat tissues after human EV injection. Accordingly, human chymase is not present in the left ventricle from a naive saline-injected rat (Figure 3A). However, in the extracellular vesicle injected rat, there is punctate human chymase in endothelial cells co-stained with von Willebrand factor and within the cardiac interstitium at 4 h after injection (Figures 3B,C). Dark red staining around the vessel shows autofluorescence of the internal elastic membrane. In addition, there is diffuse myofibrillar breakdown and interstitial edema 4 and 24 h after the injection of extracellular vesicles in the same animal (Figures 4A–C).

## 4. Discussion

In cardiovascular disease, chymase has been largely associated with chronic disease states such as cardiac fibrosis, atherosclerosis, aortic aneurysm, and renal fibrosis (5). This study demonstrates an important role for chymase in mediating acute tissue injury after on- or off-pump cardiac surgery. The univariate analysis demonstrates that STS-PROM, 4-hour post-surgery PCF chymase activity and CXCL6 levels are significant predictors of ICU and total hospital length of stay. STS PROM and chymase activity are significant in both univariate and multivariate models for the prediction of total and ICU length of stay. The importance of this study to the future therapeutic management of patients recovering from open heart surgery is further expanded by the identification that there is increased chymase activity in PCF after surgery.

We have previously reported that the inflammatory marker levels in this PCF drain compartment are significantly higher than simultaneous plasma (12). Here we show that exosomes containing chymase are present in the PCF at the time of pericardial incision and in the PCF fluid from drains placed close to the heart after cardiac surgery. Chymase is rapidly neutralized in the circulation by serine protease inhibitors as demonstrated by the presence of *α*1-anti-trypsin and *α*1-anti-chymotrypsin in the PCF by mass spectrometry (Supplementary Table S4) at the time of opening the pericardium. Mast cells are a major source of chymase and other destructive proteases and can be a major source of exosomes (35). Uptake of chymase *via* exosome delivery identifies a potential role of chymase in mediating cardiac damage *via* the protected delivery system of extracellular vesicles.

As seen in Figure 1 there is low chymase activity in the PCF fluid at the time of opening the pericardium, compared to all time points after surgery. Chymase activity *in vitro* is detected only after sonication of extracellular vesicles, suggesting that extracellular vesicles protect chymase from PCF serine protease inhibitors. Injection of these same PCF extracellular vesicles into rats *in vivo* results in chymase uptake in endothelial cells, cardiomyocytes, and interstitium of the heart within 4 h after injection. There is severe myosin breakdown and cardiomyocyte dropout, further indicating *in vivo* that extracellular vesicles may provide a protected delivery vehicle for the enzyme and other destructive proteases.

We have shown a marked influx of chymase into cardiomyocytes within two hours of ischemia/reperfusion in the dog (36). There is a significant decrease in interstitial fluid chymase-mediated Ang II formation and troponin release with chymase inhibitor pretreatment (36). Studies in the pig (37), hamster (38), and mouse (3) demonstrate that early treatment with a chymase inhibitor within 24 h after coronary artery ligation (38) or within hours of ischemia/reperfusion (3, 36, 37) decreases infarct size and improves LV remodeling and systolic dysfunction. In support of a chymase connection to cardiac ischemia, the early increase in chymase activity relates to intraoperative cross-clamp and total operative time. Early peak values of chymase- and Cathepsin G-dependent angiotensin II formation in circulating mononuclear leukocytes correlate with elevations of creatine kinase (39). Taken together, these studies may explain the failure of chymase inhibition to improve LV remodeling and function in the recent CHIARA MIA 2 clinical trial, where the chymase inhibitor was started six to 12 days post-myocardial infarction (40).

Chymase has a multifunctional role in acute tissue injury and chronic remodeling in cardiovascular disease (5). Chymase activates MMP-9 and TGF-*β* leading to tissue injury and fibrosis, activates stem cell factor promoting mast cell and neutrophil infiltration, and degrades fibronectin resulting in cardiomyocyte apoptosis (5). Chymase is the major angiotensin II forming enzyme in the human heart (34) and activates IL-6, IL-1β, and IL-18 (5). In addition to mast cells as a source of chymase, there is evidence that other cells like cardiac fibroblasts and vascular endothelial cells may also produce and secrete chymase, providing a broad spectrum of chymase production in various tissues (5). We have reported chymase within cardiomyocytes in the dog (36), the rat (33, 41, 42), and human (43), uncovering a novel compartmentalization of chymase-mediated physiological actions earlier thought to be confined to the extracellular space. In the human heart, there is abundant chymase mRNA in mast cells, endothelial cells, and other interstitial cells by *in situ* hybridization, in addition to evidence of chymase chymotryptic activity within the cardiomyocyte (43). Indeed, chymase added to adult rat cardiomyocytes *in vitro* results in myosin breakdown (33). Although the resulting myosin breakdown is prevented by blockade of uptake *in vitro* (33), chymase delivery to cells *in vivo* may involve a different mechanism.

We have also reported acute and chronic heart (31) and kidney (32) injury after injection of exosomes obtained from CPB patients 30 min after cross-clamp release. A similar destructive protease-activated polymorphonuclear exosomes taken from patients with chronic obstructive lung disease exacerbation causes elastase-mediated lung damage after injection into the lungs of naïve mice (44). Extracellular vesicles carry a dynamic myriad of cargo that may be beneficial or harmful. Future studies must resolve the outcome of short-term blockade or delivery in the context of the clinical situation.

## 5. Concluding remarks

There are many other factors to consider in Systemic Inflammatory Response Syndrome during and after cardiac surgery (1). However, the multiple functions of chymase in a systemic inflammatory response as well as Ang II formation raise for the first time, chymase carried in extracellular vesicles, as a potential target in attenuating or limiting systemic organ damage after cardiac surgery. The early increase in chymase after reperfusion raises the question of whether chymase inhibition within four hours can attenuate heart and other organ damage after cardiac surgery, especially in high-risk patients.

## Data Availability

The original contributions presented in the study are included in the article/**Supplementary Material**, further inquiries can be directed to the corresponding author/s.
