# Supplementary material for: Impact of early pericardial fluid chymase activation after cardiac surgery
Source: Front Cardiovasc Med. 2023 Apr 12;10:1132786. doi: 10.3389/fcvm.2023.1132786 (PMC10230304; doi:10.3389/fcvm.2023.1132786)

Supplemental Table 1. Biomarkers in PCF (4 hours after surgery) for on and off pump surgery						
	CABG ON/OFF	N	Mean	Std. Deviation	p-value	FDR adjusted p-value
Chymase	Off	6	3.787717	3.1918560	.919	.949
	On	8	3.951648	2.6882206		
Myeloperoxidase (37)	Off	7	497.9671	446.75294	.494	.751
	On	9	723.8467	751.26205		
GDF-15 (28)	Off	7	.8143	.45537	.216	.696
	On	9	1.1189	.47414		
ADAMTS13 (13)	Off	7	81.9400	30.33641	.197	.696
	On	9	127.4267	84.19046		
sVCAM-1 (74)	Off	7	131.9786	56.44534	.177	.696
	On	9	197.5300	110.65211		
sICAM-1 (35)	Off	7	32.5614	9.94957	.348	.696
	On	9	40.1956	18.74574		
SAA (76)	Off	7	24340.09	61365.425	.289	.696
	On	9	2010.13	2511.930		
sP-Selectin (47)	Off	7	.8114	2.14684	.241	.696
	On	9	8.3300	16.00118		
NGAL (56)	Off	7	90.4429	29.27328	.224	.696
	On	9	128.0944	73.47377		
CXCL6 (21)	Off	7	1539.0414	3484.16835	.342	.696
	On	9	404.4133	212.95206		
CXCL16 (25)	Off	7	4358.3657	2868.10515	.796	.948
	On	9	4081.9511	1186.13920		
Endocan-1 (38)	Off	7	146.0914	156.23068	.160	.696
	On	9	496.7778	604.74175		
FABP3 (45)	Off	7	56100.75	21453.476	.340	.696
	On	9	75790.29	48921.374		
FABP4 (46)	Off	7	336098.32	341693.244	.336	.696
	On	9	516262.28	371597.072		
PIGF (52)	Off	7	143.9943	176.68526	.722	.915
	On	9	121.2033	61.42549		
OSM (57)	Off	7	1.9800	2.21506	.932	.949
	On	9	1.8833	2.17747		
HMMP-3 (5)	Off	7	9479.5714	6413.84048	.823	.948
	On	9	10514.3856	10548.73936		

MMP-1 (1)	Off	7	194.3171	216.76063	.380	.722
	On	9	352.3889	417.38027		
MMP-2 (3)	Off	7	65863.1086	40940.96352	.592	.865
	On	9	75425.9711	28924.85406		
MMP-7 (7)	Off	7	7448.4086	8940.32430	.799	.948
	On	9	6578.1100	4188.52575		
MMP-9 (27)	Off	7	3381.2043	1562.43241	.236	.696
	On	9	7192.4989	7962.69365		
MMP-10 (29)	Off	7	123.3571	51.87925	.449	.743
	On	9	157.1578	104.76107		
IL-10 (27)	Off	5	49.4340	43.57148	.142	.696
	On	9	13.6178	16.49026		
IL-6 (57)	Off	6	529.1500	678.21042	.170	.696
	On	8	84.8887	86.88421		
IFNy (25)	Off	6	.7583	1.85753	.163	.696
	On	8	3.7375	4.60545		
TNFa (75)	Off	6	24.24833	4.487148	.227	.696
	On	9	18.20778	10.966256		
VEGF (78)	Off	6	43.1400	105.67099	.703	.915
	On	8	24.8450	70.27227		
IFNa2 (22)	Off	6	11.0250	10.30614	.490	.751
	On	8	6.8288	11.34417		
sCD40L (38)	Off	6	1395.1550	1694.57589	.293	.696
	On	8	2841.1750	2849.89433		
IL-15 (37)	Off	6	59.3800	39.55701	.434	.743
	On	9	44.2711	32.68145		
MCP-1 (67)	Off	6	4131.2167	2419.89059	.949	.949
	On	9	4228.9722	3052.25203		
G-CSF (18)	Off	6	362.9350	599.46987	.172	.696
	On	9	76.6256	67.80091		
IL-12(p70) (33)	Off	6	.2850	.69810	.721	.915
	On	9	.1711	.51333		
IP-10 (65)	Off	6	4044.1833	3494.03202	.336	.696
	On	9	2610.8611	2102.77963		
MDC (30)	Off	6	430.1567	218.10651	.920	.949
	On	9	441.1689	194.01459		
MIP-1b (73)	Off	6	54.9383	20.45821	.905	.949
	On	9	53.1444	31.91880		
MIP-1a (72)	Off	6	19.80333	13.616331	.616	.867

	On	9	16.58111	10.684329		
IL-8 (63)	Off	6	106.89667	172.482028	.450	.743
	On	8	50.74375	95.377610		

Independent Samples Effect Sizes

		Standardizer[a]	Point Estimate	95% Confidence Interval	
				Lower	Upper
Chymase	Cohen's d	2.9086858	-.056	-1.114	1.004
	Hedges' correction	3.1077634	-.053	-1.043	.939
	Glass's delta	2.6882206	-.061	-1.118	1.000
Myeloperoxidase (37)	Cohen's d	638.78716	-.354	-1.344	.649
	Hedges' correction	675.75950	-.334	-1.270	.613
	Glass's delta	751.26205	-.301	-1.290	.707
GDF-15 (28)	Cohen's d	.46619	-.653	-1.659	.374
	Hedges' correction	.49317	-.618	-1.568	.353
	Glass's delta	.47414	-.642	-1.660	.411
ADAMTS13 (13)	Cohen's d	66.66872	-.682	-1.690	.348
	Hedges' correction	70.52743	-.645	-1.597	.329
	Glass's delta	84.19046	-.540	-1.546	.497
sVCAM-1 (74)	Cohen's d	91.44381	-.717	-1.727	.317
	Hedges' correction	96.73648	-.678	-1.633	.300
	Glass's delta	110.65211	-.592	-1.604	.452
sICAM-1 (35)	Cohen's d	15.59576	-.490	-1.485	.523
	Hedges' correction	16.49842	-.463	-1.404	.494
	Glass's delta	18.74574	-.407	-1.402	.612
SAA (76)	Cohen's d	40217.952	.555	-.463	1.554
	Hedges' correction	42545.725	.525	-.437	1.469
	Glass's delta	2511.930	8.890	4.511	13.262
sP-Selectin (47)	Cohen's d	12.17713	-.617	-1.620	.406
	Hedges' correction	12.88193	-.584	-1.532	.384
	Glass's delta	16.00118	-.470	-1.470	.557
NGAL (56)	Cohen's d	58.75415	-.641	-1.645	.385
	Hedges' correction	62.15478	-.606	-1.555	.364
	Glass's delta	73.47377	-.512	-1.516	.520
CXCL6 (21)	Cohen's d	2286.59702	.496	-.517	1.492
	Hedges' correction	2418.94288	.469	-.488	1.410
	Glass's delta	212.95206	5.328	2.572	8.053
CXCL16 (25)	Cohen's d	2080.72055	.133	-.858	1.119
	Hedges' correction	2201.15050	.126	-.811	1.058

	Glass's delta	1186.13920	.233	-.768	1.220
Endocan-1 (38)	Cohen's d	468.44338	-.749	-1.762	.289
	Hedges' correction	495.55640	-.708	-1.665	.273
	Glass's delta	604.74175	-.580	-1.590	.463
FABP3 (45)	Cohen's d	39558.200	-.498	-1.494	.515
	Hedges' correction	41847.787	-.471	-1.412	.487
	Glass's delta	48921.374	-.402	-1.397	.616
FABP4 (46)	Cohen's d	359086.212	-.502	-1.498	.512
	Hedges' correction	379869.749	-.474	-1.416	.484
	Glass's delta	371597.072	-.485	-1.486	.544
PIGF (52)	Cohen's d	124.63972	.183	-.810	1.170
	Hedges' correction	131.85373	.173	-.766	1.106
	Glass's delta	61.42549	.371	-.644	1.364
OSM (57)	Cohen's d	2.19366	.044	-.945	1.031
	Hedges' correction	2.32063	.042	-.893	.975
	Glass's delta	2.17747	.044	-.945	1.031
HMMP-3 (5)	Cohen's d	9012.02100	-.115	-1.101	.876
	Hedges' correction	9533.62740	-.109	-1.041	.828
	Glass's delta	10548.73936	-.098	-1.084	.894
MMP-1 (1)	Cohen's d	345.95225	-.457	-1.451	.553
	Hedges' correction	365.97560	-.432	-1.372	.523
	Glass's delta	417.38027	-.379	-1.372	.637
MMP-2 (3)	Cohen's d	34589.58599	-.276	-1.265	.721
	Hedges' correction	36591.59525	-.261	-1.195	.682
	Glass's delta	28924.85406	-.331	-1.321	.680
MMP-7 (7)	Cohen's d	6654.35612	.131	-.860	1.117
	Hedges' correction	7039.50333	.124	-.813	1.056
	Glass's delta	4188.52575	.208	-.791	1.194
MMP-9 (27)	Cohen's d	6105.51912	-.624	-1.628	.400
	Hedges' correction	6458.90021	-.590	-1.539	.378
	Glass's delta	7962.69365	-.479	-1.479	.550
MMP-10 (29)	Cohen's d	86.16752	-.392	-1.384	.613
	Hedges' correction	91.15481	-.371	-1.308	.579
	Glass's delta	104.76107	-.323	-1.313	.687
IL-10 (27)	Cohen's d	28.53262	1.255	.033	2.435
	Hedges' correction	30.48546	1.175	.031	2.279
	Glass's delta	16.49026	2.172	.626	3.651
IL-6 (57)	Cohen's d	442.78370	1.003	-.145	2.116
	Hedges' correction	473.08892	.939	-.136	1.980

	Glass's delta	86.88421	5.113	2.275	7.917
IFNy (25)	Cohen's d	3.71622	-.802	-1.892	.319
	Hedges' correction	3.97057	-.750	-1.770	.298
	Glass's delta	4.60545	-.647	-1.736	.483
TNFa (75)	Cohen's d	9.041539	.668	-.408	1.720
	Hedges' correction	9.608616	.629	-.384	1.618
	Glass's delta	10.966256	.551	-.532	1.602
VEGF (78)	Cohen's d	86.79436	.211	-.855	1.268
	Hedges' correction	92.73478	.197	-.801	1.187
	Glass's delta	70.27227	.260	-.816	1.318
IFNa2 (22)	Cohen's d	10.92365	.384	-.693	1.446
	Hedges' correction	11.67130	.360	-.649	1.353
	Glass's delta	11.34417	.370	-.718	1.433
sCD40L (38)	Cohen's d	2436.03539	-.594	-1.666	.502
	Hedges' correction	2602.76368	-.556	-1.559	.470
	Glass's delta	2849.89433	-.507	-1.581	.599
IL-15 (37)	Cohen's d	35.48391	.426	-.628	1.464
	Hedges' correction	37.70943	.401	-.591	1.377
	Glass's delta	32.68145	.462	-.608	1.506
MCP-1 (67)	Cohen's d	2825.83265	-.035	-1.067	.999
	Hedges' correction	3003.06620	-.033	-1.004	.940
	Glass's delta	3052.25203	-.032	-1.064	1.002
G-CSF (18)	Cohen's d	375.56074	.762	-.324	1.822
	Hedges' correction	399.11556	.717	-.305	1.714
	Glass's delta	67.80091	4.223	1.924	6.476
IL-12(p70) (33)	Cohen's d	.59127	.193	-.847	1.225
	Hedges' correction	.62836	.181	-.797	1.152
	Glass's delta	.51333	.222	-.823	1.254
IP-10 (65)	Cohen's d	2723.32879	.526	-.536	1.569
	Hedges' correction	2894.13340	.495	-.504	1.476
	Glass's delta	2102.77963	.682	-.421	1.747
MDC (30)	Cohen's d	203.61833	-.054	-1.086	.980
	Hedges' correction	216.38908	-.051	-1.022	.922
	Glass's delta	194.01459	-.057	-1.088	.978
MIP-1b (73)	Cohen's d	28.07020	.064	-.971	1.096
	Hedges' correction	29.83074	.060	-.913	1.031
	Glass's delta	31.91880	.056	-.979	1.088
MIP-1a (72)	Cohen's d	11.897839	.271	-.772	1.304
	Hedges' correction	12.644060	.255	-.727	1.227

	Glass's delta	10.684329	.302	-.751	1.336
IL-8 (63)	Cohen's d	133.050263	.422	-.658	1.485
	Hedges' correction	142.156552	.395	-.616	1.390
	Glass's delta	95.377610	.589	-.531	1.671

a. The denominator used in estimating the effect sizes.

Cohen's d uses the pooled standard deviation.

Hedges' correction uses the pooled standard deviation, plus a correction factor.

Glass's delta uses the sample standard deviation of the control group.

Supplemental Table 2. Zero Truncated Poisson Regression Relating Pericardial Fluid Markers (4 hours after surgery) and STS-PROM Score to Hospital Length of Stay for All Variables.

Model	Coefficient	SE	p-value	AIC/AICC/BIC
Univariate Analysis				
STS-PROM	0.093	0.009	<.0001	156.6/157.7/159.4
Troponin-1 (µg/ml)	-0.0001	0.0002	.53	177.2/177.9/181.3
BNP (pg/ml)	-0.0009	0.0006	.16	178.0/178.8/179.9
Chymase (fmol/ml/min)	0.127	0.014	<.0001	117.2/117.7/119.8
TNF-α (pg/ml)	0.0026	.0015	.0026	200.1/200.7/202.6
Myeloperoxidase (pg/mL)	0.0001	0.00002	<.0001	156.8/157.6/158.6
Interleukin-8 (pg/mL)	0.00001	9.01E-6	.26	168.5/169.3/170.1
NGAL (pg/mL)	6.3E-6	8.1E-6	.44	216.7/217.2/219.3
CXCL6 (pg/ml)	0.009	0.0001	<.0001	179.2/179.7/181.9
Multivariate Analysis				
STS-PROM +<br>Troponin-1 (µg/ml)	0.09<br>7.21×10^{-6}	0.009<br>0.0001	<.0001<br>.98	108.5/109.7/112.0
STS-PROM +<br>BNP (pg/ml)	0.06<br>0.002	0.02<br>0.0007	.0001<br>.01	88.9/90.4/92.1
STS-PROM +<br>Chymase (fmol/ml/min)	0.051<br>0.068	0.021<br>0.033	.0132<br>.04	72.7/74.7/75.0
STS-PROM +<br>TNF-α (pg/ml)	0.092<br>0.001	0.009<br>0.002	<.0001<br>.05	108.7/109.9/112.2

STS-PROM + Myeloperoxidase (pg/mL)	0.09<br>0.00002	0.009<br>0.00006	<.0001<br>.73	81.05/82.90/83.55
STS-PROM + Interleukin-8 (pg/mL)	0.09<br>0.00003	0.009<br>0.00005	<.001<br>.52	74.17/76.35/76.30
STS-PROM + NGAL (pg/mL)	0.09<br>0.00007	0.009	0.0001	114.6/115.7/118.4
STS-PROM + CXCL6 (pg/ml)	0.080<br>0.006	0.009<br>0.0002	<.0001<br>.0016	110.1/111.1/114.0

BNP – brain natriuretic peptide
CXCL – chemokine (C-X-C motif) ligand
NGAL – neutrophil gelatinase-associated lipocalin
STS-PROM – Society of Thoracic Surgeons Predicted Risk of Morbidity and Mortality
TNF – tumor necrosis factor

Supplemental Table 3. Zero Truncated Poisson Regression Relating Pericardial Fluid Markers (4 hours after surgery) and STS-PROM Score to Intensive Care Unit Length of Stay for All Variables.

Model	Coefficient	SE	p-value	AIC/AICC/BIC
Univariate Analysis				
STS-PROM	0.175	0.020	<.0001	71.3/71.8/74.0
Troponin-1 (µg/ml)	-0.0002	0.0008	.80	112.2/112.9/113.8
BNP (pg/ml)	-0.002	0.001	.12	110.1/110.9/111.9
Chymase (fmol/ml/min)	0.241	0.028	<.0001	46.7/47.2/49.3
TNF-α	0.003	0.003	.30	126.0/126.6/128.4
Myeloperoxidase (pg/mL)	0.00014	0.00005	.0016	100.7/101.6/102.4
Interleukin-8 (pg/mL)	0.00005	0.00003	.06	102.8/103.7/104.4
NGAL (pg/mL)	0.0001	0.0001	.22	124.5/125.0/127.0
CXCL6 (pg/ml)	0.002	0.0002	<.0001	105.9/106.4/108.5
Multivariate Analysis				
STS-PROM +<br>Troponin-1 (µg/ml)	0.19<br>-0.0005	0.08<br>0.001	0.02<br>0.65	49.0/50.2/52.4
STS-PROM +<br>BNP (pg/ml)	0.18<br>0.003	0.03<br>0.003	<.0001<br>0.2	49.7/51.5/52.4
STS-PROM +<br>Chymase (fmol/ml/min)	0.009<br>0.271	0.067<br>0.130	0.89<br>0.04	30.2/32.2/32.5

STS-PROM +<br>TNF-α (pg/ml)	0.20<br>-0.02	0.03<br>0.01	<.001<br>0.15	44.5/45.7/48.0
STS-PROM +<br>Myeloperoxidase (pg/mL)	0.17<br>0.00006	0.033<br>0.0001	<.0001<br>.63	39.8/41.6/42.3
STS-PROM +<br>Interleukin-8 (pg/mL)	0.15<br>0.00004	0.021<br>0.00004	<.0001<br>.35	38.5/40.5/40.8
STS-PROM +<br>NGAL (pg/mL)	0.17<br>0.00002	0.022<br>0.00006	<.0001<br>.70	46.1/47.2/49.8
STS-PROM +<br>CXCL6 (pg/ml)	0.139<br>0.001	0.022<br>0.0005	<.0001<br>.03	44.64/45.7/48.5

BNP – brain natriuretic peptide
CXCL – chemokine (C-X-C motif) ligand
NGAL – neutrophil gelatinase-associated lipocalin
STS-PROM – Society of Thoracic Surgeons Predicted Risk of Morbidity and Mortality
TNF – tumor necrosis factor

**Supplemental Table 4. Mass spectrometry of PCF (at opening of the pericardium = time 0) from on-pump patients (N = 2)**				
**Protein**	**Rep ID**	**Accession Number**	**Unique Peptide Count**	
			Patient 06	Patient 16
Serum albumin	ALBU_HUMAN	P02768	40	34
Apolipoprotein A-I	APOA1_HUMAN	P02647	32	35
Alpha-1-antitrypsin	A1AT_HUMAN	P01009	18	18
Epididymis luminal protein 213	V9HW34_HUMAN	V9HW34	15	16
Clusterin	CLUS_HUMAN	P10909	12	8
Fibrinogen alpha chain	FIBA_HUMAN	P02671	11	22
Complement C4-A	CO4A_HUMAN	P0C0L4	11	13
Apolipoprotein E	APOE_HUMAN	P02649	11	4
Lambda-chain (AA -20 to 215)	A2NUT2_HUMAN	A2NUT2	10	12
Apolipoprotein D	APOD_HUMAN	P05090	10	6
Transthyretin	TTHY_HUMAN	P02766	9	11
Epididymis luminal protein 214	V9HW68_HUMAN	V9HW68	9	8
Hemoglobin subunit beta	HBB_HUMAN	P68871	9	2
Serotransferrin	TRFE_HUMAN	P02787)	8	6
Carbonic anhydrase 1	CAH1_HUMAN	P00915	8	3
Protein AMBP	AMBP_HUMAN	P02760	6	9
Ig kappa chain V-III region HAH	KV312_HUMAN	P18135	6	4
Carbonic anhydrase 2	CAH2_HUMAN	P00918	6	2
Haptoglobin	HPT_HUMAN	P00738	5	11
Intelectin 1	Q5IWS5_HUMAN	Q5IWS5	5	7
Complement factor D preproprotein	A6XNE2_HUMAN	A6XNE2	5	6
Serum amyloid P-component	SAMP_HUMAN	P02743	5	3
Hemopexin	HEMO_HUMAN	P02790	4	8
Complement C3	CO3_HUMAN	P01024	4	7
Ig gamma-2 chain C region	IGHG2_HUMAN	P01859	4	7
Mesothelin	MSLN_HUMAN	Q13421	4	7
Ig kappa chain V-II region TEW	KV204_HUMAN	P01617	4	3
Ig kappa chain V-I region WEA	KV118_HUMAN	P01610	4	2
Metalloproteinase inhibitor 1	TIMP1_HUMAN	P01033	3	5
Complement C1q subcomponent subunit B	C1QB_HUMAN	P02746	3	4
Hemoglobin subunit alpha	HBA_HUMAN	P69905	3	3
Ig lambda chain V-III region LOI	LV302_HUMAN	P80748	3	3
Ig lambda chain V-III region SH	LV301_HUMAN	P01714	3	3
Immunoglobulin lambda-like polypeptide 5	IGLL5_HUMAN	B9A064	3	3
Inter-alpha (Globulin) inhibitor H4 (Plasma Kallikrein-sensitive glycoprotein)	B2RMS9_HUMAN	B2RMS9	3	3
Ig kappa chain V-I region Lay	KV113_HUMAN	P01605	3	2
Prostaglandin-H2 D-isomerase	PTGDS_HUMAN	P41222	3	2

Vitamin D-binding protein	VTDB_HUMAN	P02774	3	2
Apolipoprotein A-IV	APOA4_HUMAN	P06727	3	1
Extracellular superoxide dismutase [Cu-Zn]	SODE_HUMAN	P08294	3	1
Retinol-binding protein 4	RET4_HUMAN	P02753	3	1
Alpha-1-antichymotrypsin	AACT_HUMAN	P01011	2	5
Complement factor B	CFAB_HUMAN	P00751	2	4
Ig kappa chain V-I region EU	KV106_HUMAN	P01598	2	3
Ig kappa chain V-IV region Len	KV402_HUMAN	P01625	2	3
Alpha-1-acid glycoprotein 1	A1AG1_HUMAN	P02763	2	2
Apolipoprotein A-II	APOA2_HUMAN	P02652	2	2
Ig lambda chain V-I region HA	LV102_HUMAN	P01700	2	2
Ig lambda-7 chain C region	LAC7_HUMAN	A0M8Q6	2	2
Insulin-like growth factor-binding protein 6	IBP6_HUMAN	P24592	2	2
Anti-RhD monoclonal T125 kappa light chain	Q5EFE6_HUMAN	Q5EFE6	2	1
Antithrombin-III	ANT3_HUMAN	P01008	2	1
Ig kappa chain V-I region AG	KV101_HUMAN	P01593	2	1
Ig kappa chain V-I region AU	KV102_HUMAN	P01594	2	1
Ig kappa chain V-I region Mev	KV120_HUMAN	P01612	2	1
Ig kappa chain V-I region Wes	KV119_HUMAN	P01611	2	1
Ig lambda chain V region 4A	LV001_HUMAN	P04211	2	1
Ig lambda chain V-V region DEL	LV501_HUMAN	P01719	2	1
L-lactate dehydrogenase B chain	LDHB_HUMAN	P07195	2	1
Tetranectin	TETN_HUMAN	P05452	2	1
Fibrinogen gamma chain	FIBG_HUMAN	P02679	1	7
Zinc-alpha-2-glycoprotein	ZA2G_HUMAN	P25311	1	5
Complement factor I	CFAI_HUMAN	P05156	1	3
Triosephosphate isomerase	TPIS_HUMAN	P60174	1	3
V-set and immunoglobulin domain-containing protein 4	VSIG4_HUMAN	Q9Y279	1	3
14-3-3 protein zeta/delta	1433Z_HUMAN	P63104	1	2
Actin, cytoplasmic 1	ACTB_HUMAN	P60709	1	2
Alpha-1B-glycoprotein	A1BG_HUMAN	P04217	1	2
Annexin A5	ANXA5_HUMAN	P08758	1	2
Complement component C9	CO9_HUMAN	P02748	1	2
Complement factor H-related protein 1	FHR1_HUMAN	Q03591	1	2
Ig kappa chain V-III region B6	KV301_HUMAN	P01619	1	2
Ig lambda chain V-II region NIG-84	LV211_HUMAN	P04209	1	2
IGL@ protein	Q8N5F4_HUMAN	Q8N5F4	1	2
Immunoglobulin lambda-like polypeptide 1	IGLL1_HUMAN	P15814	1	2
Keratin 1	H6VRG3_HUMAN	H6VRG3	1	2
Ribonuclease pancreatic	RNAS1_HUMAN	P07998	1	2

Supplemental Figure 1. PCF protein electrophoresis for LC-MS

PCF samples obtained at opening of the pericardium (time 0) [MRP06 and MRP16] were used for LC-MS (**Supplemental Table 4**). Following BCA protein assay, 40ug of total protein was loaded per lane in triplicate for each sample and separated on a 10% Bis-tris gel. The gel was stained overnight with Colloidal Coomassie. The region corresponding to the molecular weight range between 27-34kDa (red bracket) was digested with trypsin overnight. Digests from all three lanes were combined for each patient sample prior to LC-MS analysis.

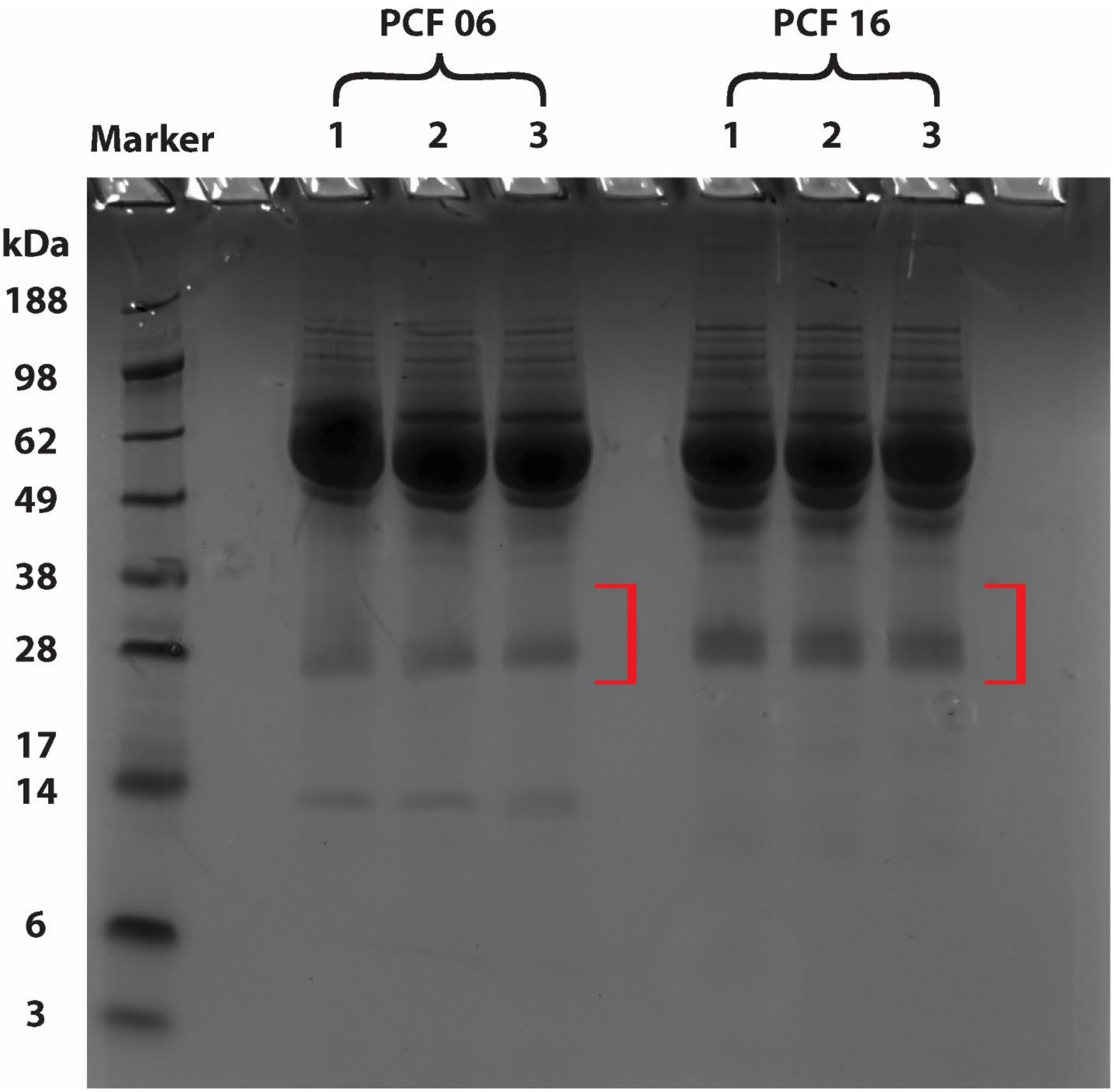

Supplemental Figure 2. Chymase expression in extracellular vesicles isolated from the PCF at opening of the pericardium (time = 0hr) during cardiac surgery.

Western blot of chymase in extracellular vesicles isolated from the pericardial fluid (at time 0) from four sternotomy on pump mitral valve repair patients (MRP08, MRP37, MRP23, MRP26). Representative western blot of human chymase (monoclonal Ab #MAB4099, R&D Systems Inc. Minneapolis, MN) in a 20uL (~$10^{8\text{-}9}$ EVs) volume of exosomes (Exos) or microvesicles (Mvs) isolated from PCF. Samples were run with a positive control of 20ng purified human skin chymase (hChymase - #BML-SE281-0010; Enzo Life Sciences, Inc. Farmingdale, NY) indicating a single band ~27-30kDa (black arrow). Note that there is significant albumin contamination in PCF extracellular vesicle isolates causing bleaching of bands between 50-75kDa (red brackets).

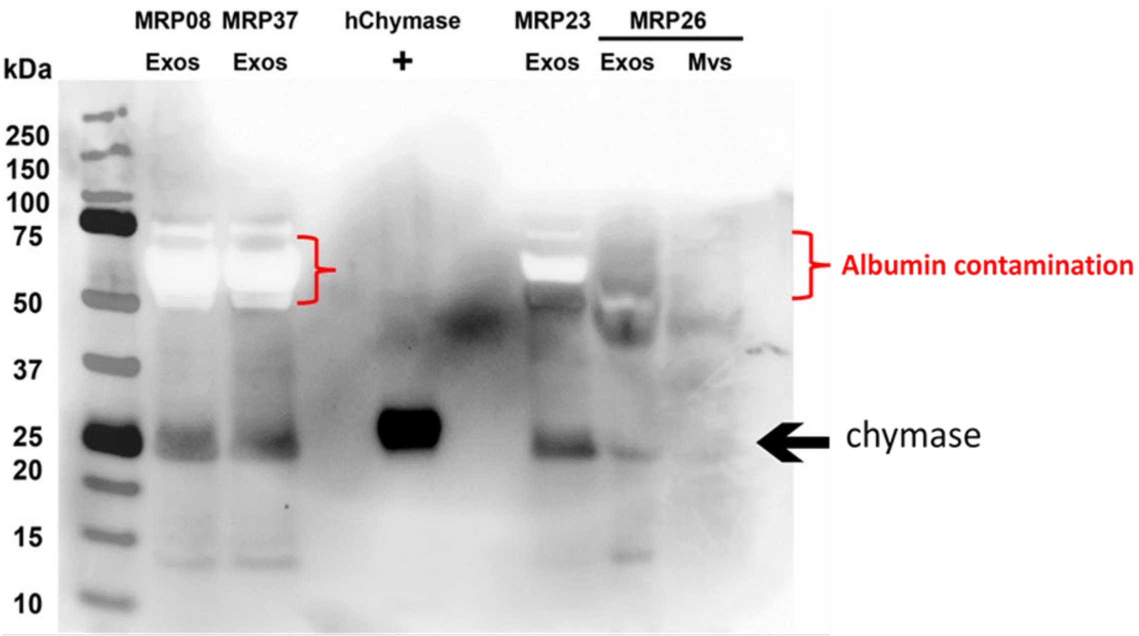

Supplement: Supplementary file 1 [file Datasheet1.pdf]
