# Supplementary figures and images for: Impact of early pericardial fluid chymase activation after cardiac surgery
Source: Front Cardiovasc Med. 2023 Apr 12;10:1132786. doi: 10.3389/fcvm.2023.1132786 (PMC10230304; doi:10.3389/fcvm.2023.1132786)

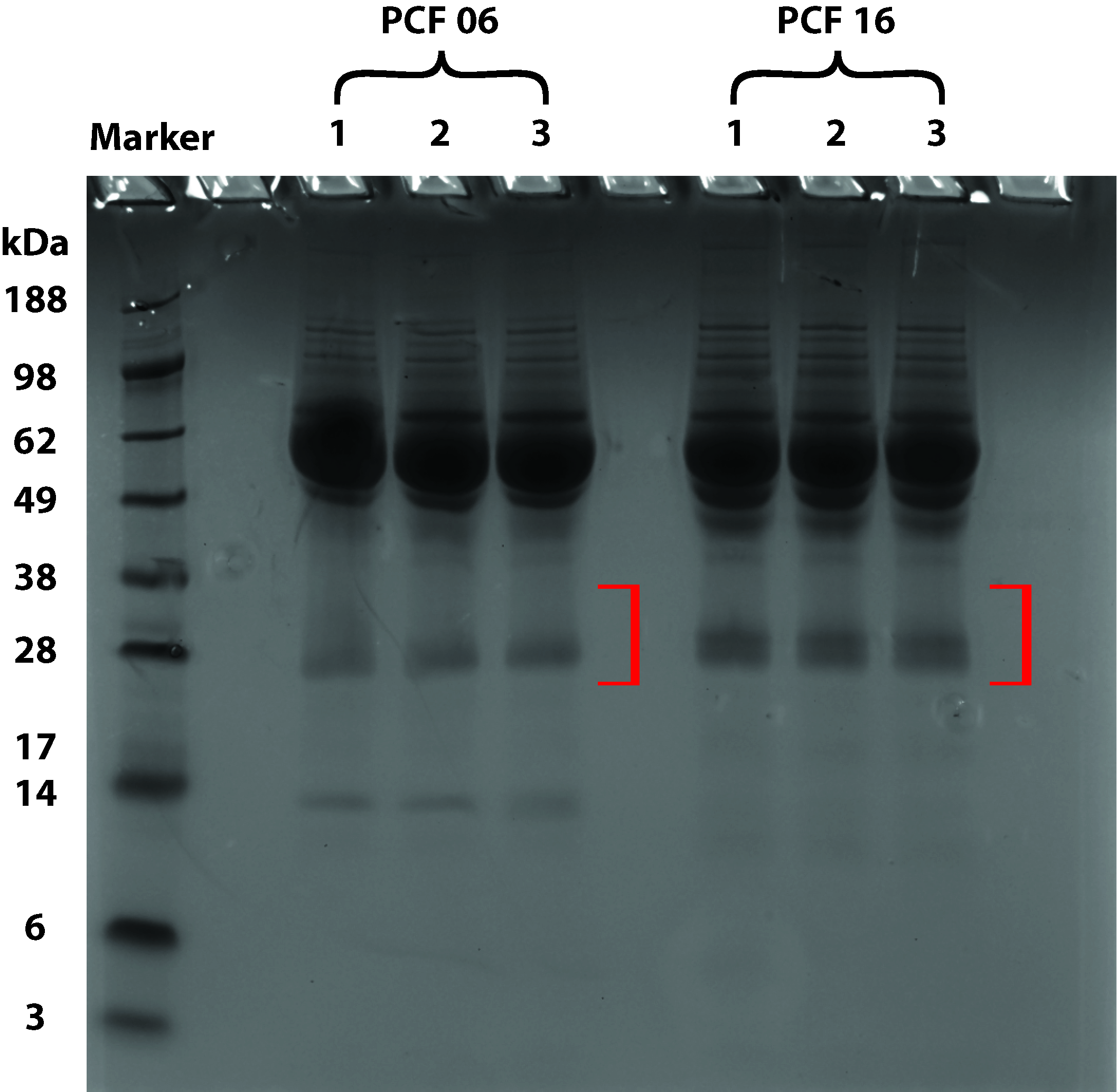

Supplement: Supplementary file 2 [file Image1.tif]

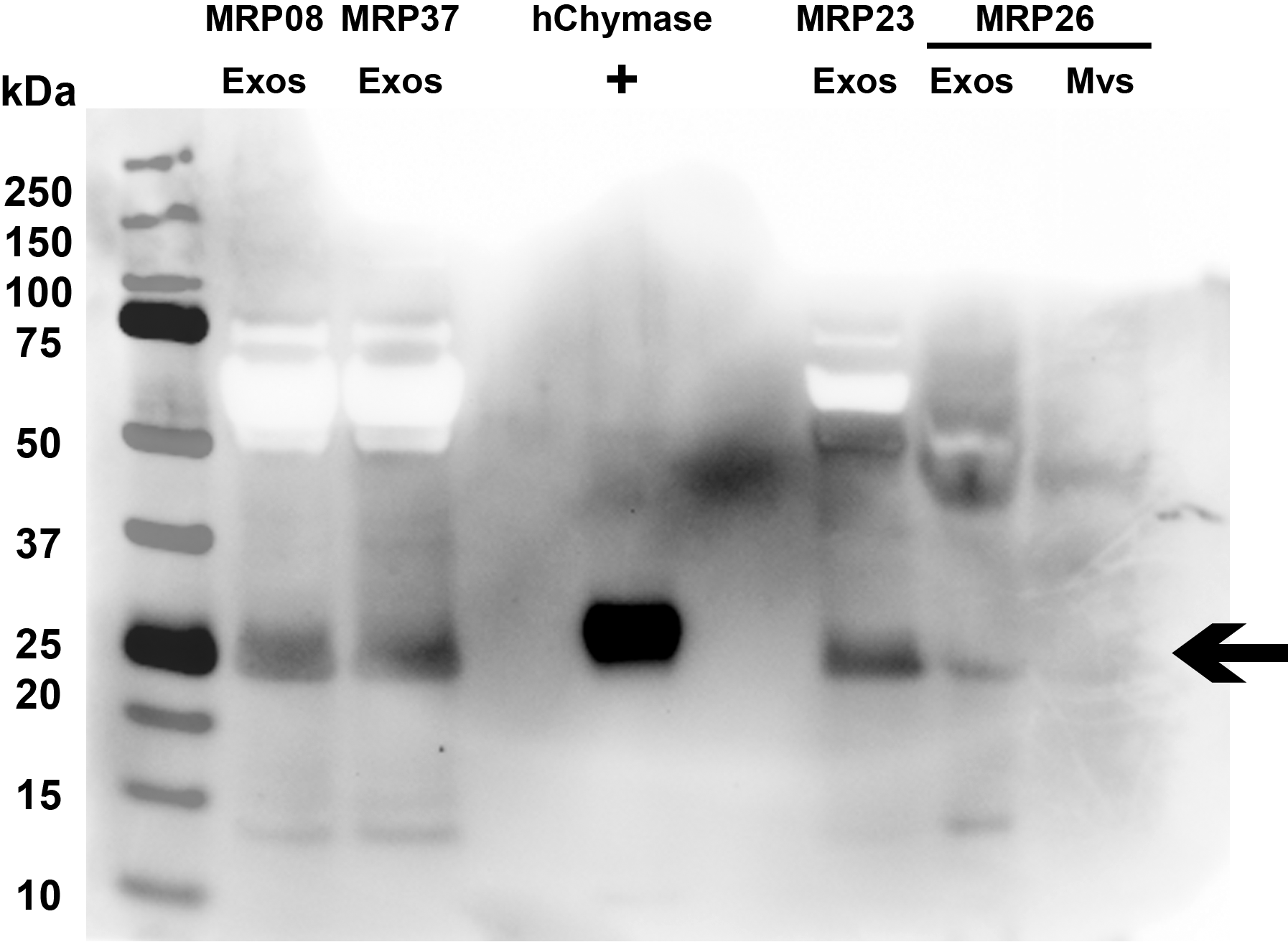

Supplement: Supplementary file 3 [file Image2.tif]
